# Best Practices for Technical Reproducibility Assessment of Multiplex Immunofluorescence

**DOI:** 10.3389/fmolb.2021.660202

**Published:** 2021-08-31

**Authors:** Caddie Laberiano-Fernández, Sharia Hernández-Ruiz, Frank Rojas, Edwin Roger Parra

**Affiliations:** Department of Translational Molecular Pathology, The University of Texas MD Anderson Cancer Center, Houston, TX, United States

**Keywords:** reproducibility, standardization, analytical evaluation, clinical application, multiplex immunofluorescence

## Abstract

Multiplex immunofluorescence (mIF) tyramide signal amplification is a new and useful tool for the study of cancer that combines the staining of multiple markers in a single slide. Several technical requirements are important to performing high-quality staining and analysis and to obtaining high internal and external reproducibility of the results. This review manuscript aimed to describe the mIF panel workflow and discuss the challenges and solutions for ensuring that mIF panels have the highest reproducibility possible. Although this platform has shown high flexibility in cancer studies, it presents several challenges in pre-analytic, analytic, and post-analytic evaluation, as well as with external comparisons. Adequate antibody selection, antibody optimization and validation, panel design, staining optimization and validation, analysis strategies, and correct data generation are important for reproducibility and to minimize or identify possible issues during the mIF staining process that sometimes are not completely under our control, such as the tissue fixation process, storage, and cutting procedures.

## Introduction

Multiplex immunofluorescence (mIF) tyramide signal amplification (TSA) is a new and useful tool for the study of cancer that combines the staining with multispectral imaging analysis technology, allows the design of mIF panels for up to six biomarkers, characterizes the co-expression of markers (cell phenotypes), and quantifies these markers overall with the use of a nuclear counterstain (DAPI) in a single slide (Parra et al., 2019; Francisco-Cruz et al., 2020b). Different mIF panels can be created using this technology to study the tissue microenvironment. The multispectral fluorescence microscope, along with the combined markers and individual fluorophores, is used to create a multispectral image that facilitates the analysis. By incorporating image analysis software, the images generated by the scanners can be easily analyzed and the cellular populations quantified (Parra et al., 2021a). mIF facilitates assessments at the cellular level of different proteins, as well as their spatial arrangement, and thus enables precision medicine in immuno-oncology, translational research, and clinical practice by elucidating the immune response of the human body to diverse tumors and showing differences in the pre- and post-treatment tissue.

Using mIF, it is possible to study the co-expression between markers to identify distinct cell populations and pathways and their relationships in different tissues and in turn to determine their roles in clinical outcomes (Parra, 2021). In that way, targetable biomarker pathways, such as PD-1/PD-L1, can be studied to verify the effect of immune therapies in the tumor microenvironment and their clinical benefit (Velcheti et al., 2014; Schalper et al., 2015; Parra et al., 2018a; Barua et al., 2018). This technology therefore has an important role in translational oncology research (Stauber et al., 2010; Steiner et al., 2014; Sood et al., 2016; Rost et al., 2017; Gorris et al., 2018) and facilitating our understanding of the disease (Blom et al., 2017; Hofman et al., 2019). mIF also has applicability for diseases other than cancer, and it is well suited for prognostication at early stages of pathogenesis, when key signaling protein levels and activities are perturbed (Dejima et al., 2021). On the clinical side, there is high demand to incorporate mIF in a Clinical Laboratory Improvement Amendments (CLIA) certified as an innovative tool for diagnosis and prognosis.

The mIF-TSA workflow starts with antibody selection, optimization, and validation and ends with a digital image analysis (Parra et al., 2020). It is important to refine, standardize, optimize, and validate the end-to-end workflow in mIF to obtain reproducible results to support large-scale multi-site trials and individual principal investigator projects and to enable their possible clinical application.

The reproducibility of results remains the cornerstone of modern science (Hewitt, 2016). Given reproducible results, considering possible technical and human problems, with adequate protocols, each laboratory or institution can proceed in the same direction, using published experiences as a reference. Pre-analytic, analytic, and post-analytic variables that may influence reproducibility, quality, and staining procedure should be considered (Rojo et al., 2009; Okoye and Nnatuanya, 2015; Rudbeck, 2015; Meyerholz and Beck, 2018). Most of the descriptions related to these variables are focused on immunohistochemistry (IHC) on the basis of a study by Engel and others, who recognized more than 60 variables in the pre-analytic stage alone (Engel and Moore, 2011) and some variables which can be considered are pre-fixation, reagent conditions, and slide preparation, but those same variables can also be applied for IF and mIF.

It was recognized over a decade ago that standardization is vital for reproducible and reliable results in IHC (Goldstein et al., 2007). Agencies such as the Biological Stain Commission, Clinical and Laboratory Standards Institute, The U.S. Food and Drug Administration, and the manufacturing sector have established guidelines, standards, and recommendations for reagents and package inserts (Taylor, 1992; Taylor, 1998; Taylor, 1999; Goldstein et al., 2007). Although all of this effort has improved the quality of IHC, most of the causative responsibility rests with the individual laboratory performing the analysis, specifically the lack of standardization and attention to quality assurance programs (Rhodes, 2003; Varma et al., 2004; Goldstein et al., 2007).

CLIA requirements for determining test performance specifications apply to all laboratory tests. All the improvements related to reproducibility can positively affect the CLIA evaluation. For IHC assays, accuracy, analytic sensitivity, and specificity are determined by analytic assay validation, which is theoretically achieved by testing a validation tissue set against a gold standard (Fitzgibbons et al., 2014). In the last year, we saw an increase in the use of this technique but the requirement aspects to be reproducible are not well established between the different centers and research groups. There are also few manuscripts about mIF reproducibility (Akturk et al., 2021; Taube et al., 2021) which have been published; thus, it is important to compare the results directly.

In the present article, we review and describe the difficulties in the reproducibility of the main workflow-related steps of the mIF technique and how to optimize the process.

## Pre-Analytic Evaluation

To develop a reproducible mIF imaging platform, several technical requirements must be met: 1) rigorous tissue quality controls, 2) a balanced multiplex assay staining format, 3) the ability to quantitate multiple markers in a defined region of interest (considering a minimum number of areas selected), and 4) experimental reproducibility, both internally and across different laboratories (Shipitsin et al., 2014).

For all these considerations, the IHC and mIF staining and imaging protocols must be standardized, automated, and validated. Being able to adapt IHC workflows in mIF without extensive re‐optimization saves time and avoids human error, making it useful for translational research and future clinical applications (Tumeh et al., 2014; Giraldo et al., 2018; Tan et al., 2020).

## Antibody Selection, Optimization, and Controls Guiding Reproducibility

The staining protocol for mIF can begin with the selection of the antibodies and their optimization by IHC or IF according to the experience and confidence of the pathologist, especially when starting with IF instead of IHC (Carvajal-Hausdorf et al., 2015). In that way, the antibody selection for mIF panel design can be considered the first step for developing a panel and needs to be done by a multidisciplinary team, including pathologists, oncologists, and immunologists. Some antibodies can be selected because of their clinical implications, while other antibodies, such as those targeting immune checkpoint markers (Francisco-Cruz et al., 2020a) may be selected to answer specific scientific or research questions. Then, choosing the correct antibody’s clones and their optimization by IHC or IF is crucial to detect specific epitopes. In parallel, the selection of correct controls, negative or positive, is essential to the valid interpretation of the staining (Engel and Moore, 2011), and it is one aspect by which methods can be systematically assessed in consecutive multiplexed assays to confirm reproducibility (Canadian Association of Pathologists-Association canadienne des pathologistes National Standards Committee et al., 2010; Stack et al., 2014). For antibody selection, each antibody’s clonality must be considered regarding its advantages and disadvantages (Table 1). Monoclonal antibodies are often preferred for IHC and IF because of their higher specificity and reproducibility and lower background and lot-to-lot variability. They are usually generated against unique peptides of the target antigen, located in regions that are less affected by formalin fixation. In contrast, polyclonal antibodies bind to different epitopes on the same protein and are obtained from experimental animals through repetitive stimulation of the antigen. Finally, recombinant antibodies, produced by recombinant DNA technology, should also be considered.

**TABLE 1:** Advantage and disadvantages of polyclonal and monoclonal antibodies.

Antibody’s type			
—	Polyclonal	Monoclonal	Recombinant
Advantage	Low cost to produce	Homogeneity is conserved between batches to ensure reproducible results	Improved reproducibility and control
	Quick turnaround time from antigen preparation to antibody harvesting	High specificity for single epitope	Antibodies can be produced rapidly
	Ability to detect multiple epitopes on an antigen	Less background	No host animals are need
	High affinity and sensitivity to detect low quantity proteins	Specificity of monoclonal antibodies make them efficient	Easier isotype conversion
	Preferred for detection of denatured proteins	Cross-reactivity with other molecules is reduced	
Disadvantage	Higher tolerance for differences in antigen	Significantly more expensive to produce	High cost to develop and produce
	Variability in each batch	Require more specialized training to create and have a much longer turnaround time	High degree of technical skills of the professionals is required
	Non-specific antibody	Cover only one epitope	
	Multiple epitopes cause high chance of cross-reactivity resulting in higher background	More sensitive to buffer conditions	

**FIGURE 1:**
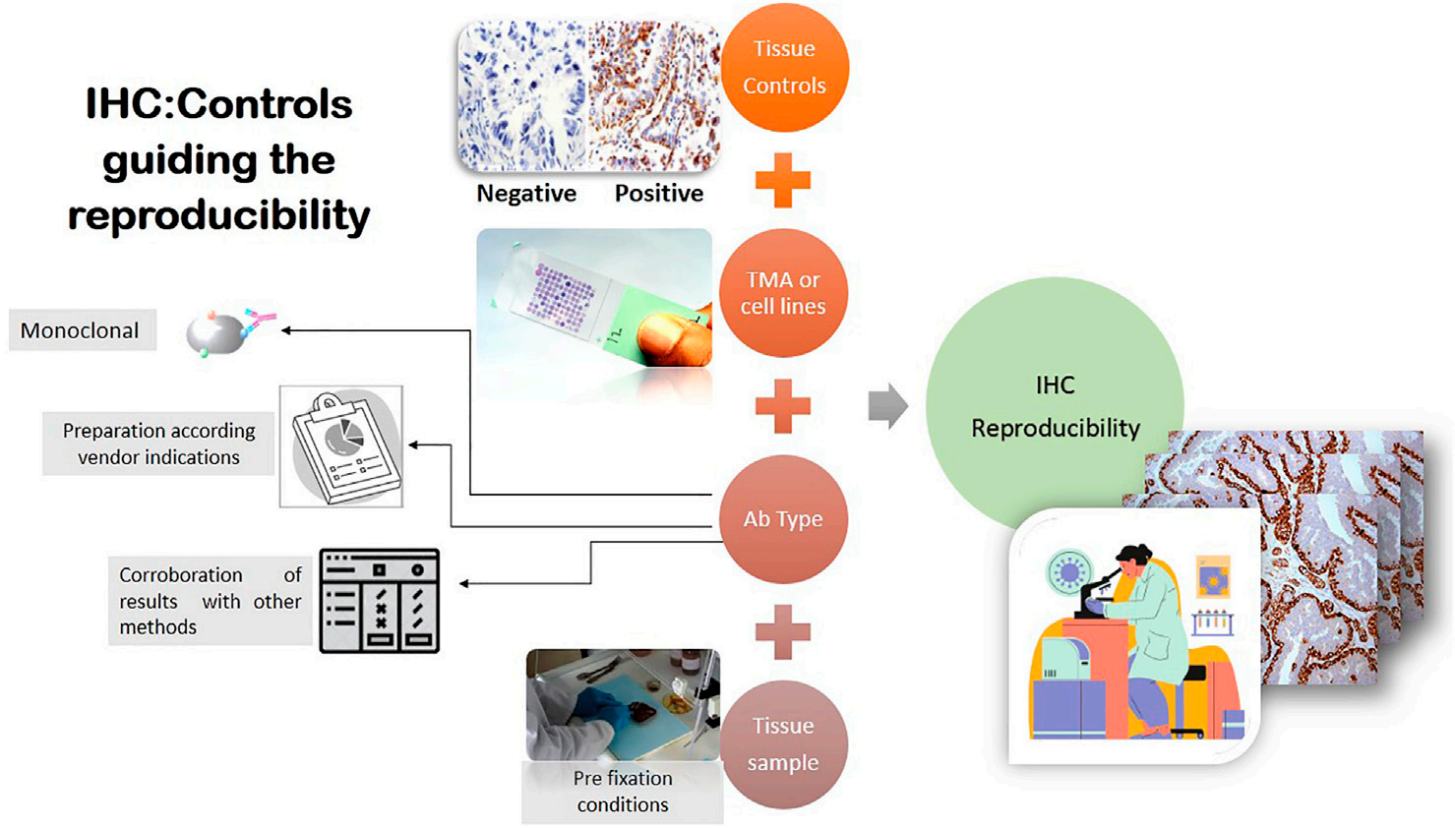
One of the most important steps in obtaining reproducibility in mIF is evaluating antibodies in IHC. Different factors must be considered to provide the best results; studies of tissue controls, TMAs, or cell lines are needed to analyze the staining of each marker using vendors’ instructions or via corroboration by other methods, such as Western blot analysis. The pre-analytical process can also affect the marker expression results; all of these factors together are part of IHC reproducibility.

**FIGURE 2:**
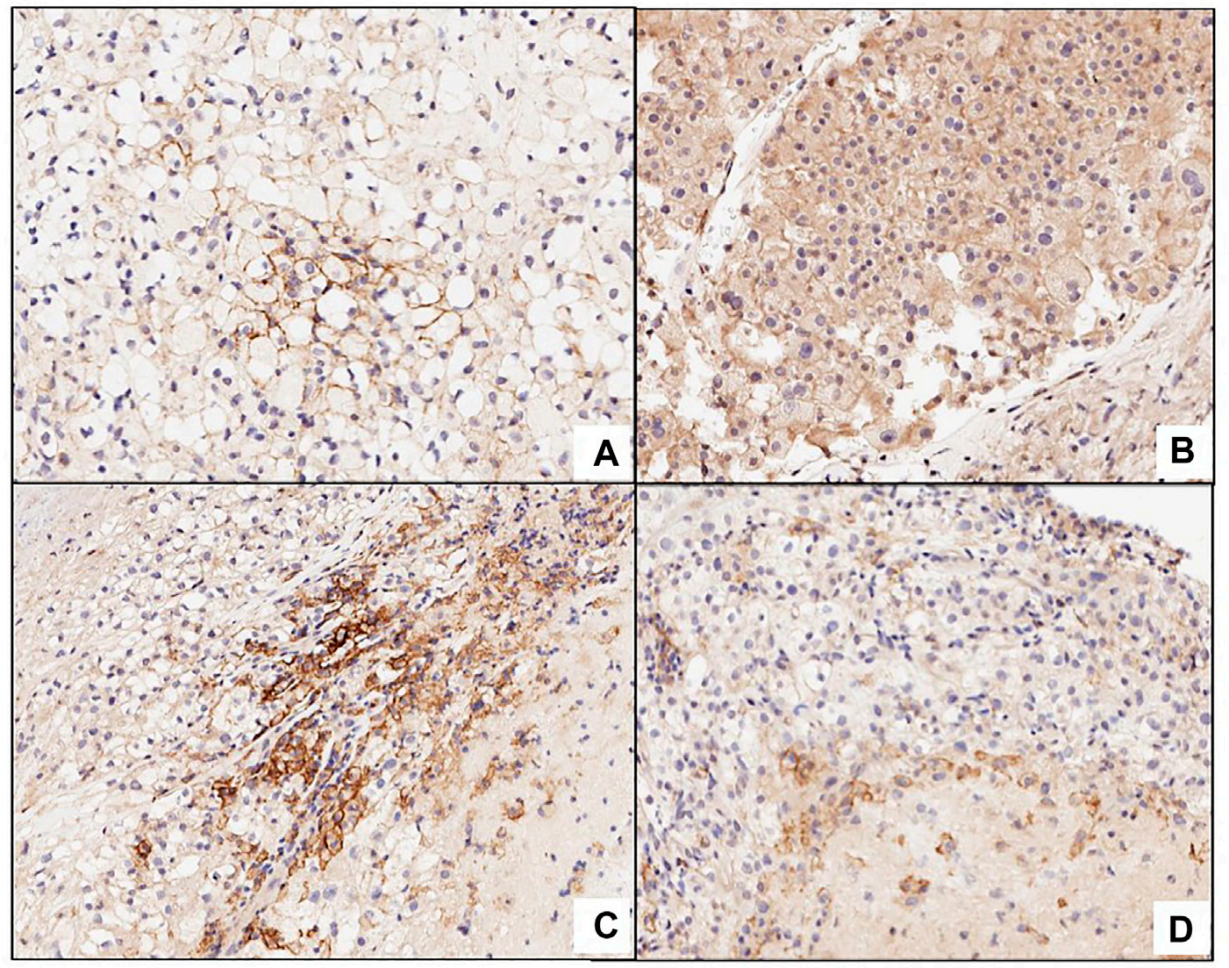
Picture **(A)** shows a positive membrane staining in PD-L1 in clear renal cell carcinoma. Picture **(B)** expresses cytoplasmic staining that is not considered positive in the evaluation. C and D are false positive because both are expressing the marker in inflammatory cells or macrophages.

Another aspect to evaluate is the potential impact of antibody sensitivity and specificity during the optimization process considering the antibodies must be verified by the user (Taylor, 1999). Besides, in the optimization process, staining intensity can be modified according to the results of a pre-analytic study, which may be affected by methodological variables such as tissue fixation, antibody specificity and dilution, antigen retrieval duration and type, and detection systems (Ng et al., 2018). For this reason, it is crucial to compare samples using external or internal control. While cell lines are useful for testing individual markers and defining their expression level, they are not completely appropriate to use as positive controls; the most rigorous are tissue controls (Hewitt et al., 2014), which can contain multiple proteins, unlike pure cell line preparations. In addition, negative controls are used to demonstrate that the reaction visualized is a result of the interaction of the epitope of the target molecule and the paratope of the antibody or affinity reagent, demonstrating the specificity of the antibody (Hewitt et al., 2014) during the run staining. Although antibodies must be prepared according to the vendors’ instructions, the experience of laboratory members, under pathologist supervision, is important to determine optimal staining conditions and correct marker expression as part of quality control Figure 1). In this regard, the primary antibody should be titrated to an appropriate concentration that retains the specificity of the stain while removing any background signal or non-specific staining of the tissue. Antibodies that are prepared at a too high concentration can result in off-target staining (Anagnostou et al., 2010; Toki et al., 2017); an optimal concentration results in better accuracy and reproducibility (Toki et al., 2017; Taube et al., 2020). The adequate expression must be tested because some markers are able to stain more than one compartment of cells or other types of cells (e.g., PD-L1 could have cytoplasmic expression, but only membrane expression is considered positive staining, and it could be expressed in inflammatory cells besides the malignant cells) (Parra and Hernández Ruiz, 2021a) **(** Figure 2 **)**.

## Strategies for Antibody Validation

One of the key factors for mIF panel reproducibility is to use antibodies that have been thoroughly optimized and validated for their application in research studies or for clinical applications. After antibody optimization by IHC or IF in control tissues, a good practice is applying those antibodies in a set of different tissues and organs including different common cancer types contained in tissue microarrays (TMAs) for quantitative measurement and antibody testing and validation. Although the construction of TMAs is often expensive for some laboratories (Taube et al., 2020), it is highly recommended to test the antibodies that will be integrated with an mIF panel in at least a set of cases for validation purposes, as a minimum requirement (Parra et al., 2017). The International Working Group for Antibody Validation proposed in total five different “pillars” to use for antibody validation with 1) genetic, 2) orthogonal, 3) independent antibody strategies, 4) expression of tagged proteins, and 5) immunocapture followed by mass spectrometry. It is recommended to consider at least one of these pillars as a minimum criterion for claiming that a selected protein has been adequately valid for a particular application (Uhlen et al., 2016). The most common and mainstay strategies are the orthogonal and the independent antibody strategy (Sivertsson et al., 2020). In the case of orthogonal validation (the most common), for an mIF panel validation, we use a non-antibody-based method to identify any effects or artifacts that are directly related to the antibody or panel in question (Sivertsson et al., 2020). Depending on the antibodies targeted in a panel, non-antibody-based methods can include mining previously published results. Overall, it is possible studying expression analysis *via* genomics, transcriptomics, and proteomics techniques; or employing other established antibody-independent methods such as *in situ* hybridization or RNA sequencing. This strategy can also be used to ensure that any antibody validation performed in-house uses the most relevant biological models for the targets of interest. Although immunostaining techniques that are established in a lab, such as Western blot (Parra et al., 2018b), in positive and negative cell lines (Bordeaux et al., 2010) for research antibodies, can help provide a quick visual indication of antibody specificity (Parra et al., 2020; Parra and Hernández Ruiz, 2021b), it is always important and recommended that the antibody’s data generated be supported by orthogonal testing. One way of achieving this is to mine publicly available databases (e.g., CCLE, BioGPS, Human Protein Atlas, DepMap Portal, COSMIC) for genomic and transcriptomic profiling information to clarify whether observed immunostaining results are relevant or are instead due to antibody-related artifacts (Cell Signaling, 2019; Ghandi et al., 2019; Broad Institute, 2021; COSMIC, 2021; The Human Protein Atlas, 2021).

About the independent antibody strategy, this is characterized by the use of independent antibodies, defined as a similar expression pattern determined by an independent antibody targeting a non-overlapping region of the similar protein (Sivertsson et al., 2020). Two or more independent antibodies that acknowledge a similar target may be used to assess antibody specificity in a range of assays. This approach requires that the expression patterns generated by the two antibodies correlate within a given application environment, which means that the two antibodies are able to bind to totally different regions of the protein and thus have different epitopes, minimizing the likelihood of off-target binding to a similar unrelated protein (Uhlen et al., 2016). Although diverse techniques can be used for antibody validation according to the necessities of the studies as described above, it is important to consider, when choosing one, its advantages and disadvantages, which are described in Table 2

**TABLE 2:** Strategies and methods for antibody/multiplex immunofluorescence panel validation.

Strategy	Method	Advantage	Disadvantage
Genetic	*In situ* hybridization (ISH), CRISPR/CAS9 or siRNA/shRNA, Western blot	- Novel genes in spatial contest	- Limited co-expression
		- The use of genome editing techniques is preferred	- Need functional knockdown reagents
		- Provide a direct link between the gene, the target protein, and its detection by the antibody	- Cannot be used for human tissue samples and body fluids (plasma and serum)
		- Useful for examining antibody specificity for proteins that come from related genes	- Time-consuming
Orthogonal	Fluorescent *in situ* hybridization (FISH), quantitative PCR, RNA-seq, Western blot	- Expression of the target protein is compared with an antibody-independent method	- Limited probes and parameters
		- Co-expression in spatial context	- Need differential expression of target protein
Independent antibody	Immunofluorescence imaging, Immunohistochemistry, Western blot	- Co-expression can be in spatial context	- Limited parameters
		- The data generated using several antibodies (different epitopes) in the same protein is compared	- Need antibodies with different epitopes
Tagged protein expression	Immunohistochemistry, Western blot	- Novel target in spatial context	- Limited co-expression
		- Tagged proteins should be expressed at endogenous levels	- Overexpression of the target protein might mask the detection of off-target binding events
			- Limitations of this method are similar to those of the genetic approaches
			- Avoid potential artifacts introduced by the tag itself
Immunocapture followed by mass spectrometry	Immunoprecipitation, chromatin immunoprecipitation	- Fast, easily co-expression	- Many proteins have similar size
		-This is one of the best methods for identifying off-target protein binding	- Difficulty in distinguishing direct interactors with the antibody versus proteins that form relevant complexes with the target protein
			- Some of the antibodies validated still do not perform in immunofluorescence assays

## mIF Optimization and Control Selection

mIF panel development is essentially the consolidation of a single IF protocol in a multiplex protocol (Taube et al., 2020); it should ideally be performed using tissues with a full range of known expression patterns for the targets of interest, using the same positive and negative controls as described above for antibody optimization and validation. Careful project design is mandatory, as well as choosing correct, reliable, and very well optimized antibodies to create a panel; other important variables for optimizing results include fresh tissue sections and regular or thin tissue slices (maximum, 4 µm) and adequately charged slides to avoid tissue detachment. It is important to use very well-known control tissues during each run of staining to detect possible errors in the mIF panel; for example, human reactive tonsil is frequently used during mIF optimization because we know the exact distribution of its different cell populations (Parra et al., 2017). Although it has been demonstrated that we can design panels containing up to eight antibody targets (Parra et al., 2021b), the complexity of handling will increase with the number of markers introduced in a panel. For the pre-analytical step, it is also necessary to consider individual marker signals; the subcellular location of the targets’ expression (nuclear, membrane, and cytoplasmic); optimization of antigen retrieval conditions (pH and temperature); reagent titration (e.g., primary antibody, secondary antibody, and fluorophores); incubation conditions (time and temperature); and blocking of non-specific binding, following similar rigor to that described in the antibody validation.

Besides the factors mentioned before, two important aspects remain. First, because TSA reagents covalently bind to sites surrounding the antigen, they can potentially inhibit the binding of a subsequent primary antibody through steric hindrance. This phenomenon is considered an umbrella effect and tends to occur in situations where multiple markers reside in a single cell compartment, such as a CD3^+^ CD8^+^ PD-1+ T cell, where all three markers are expressed on the cell membrane. It is conceivable that if CD3 and/or CD8 comes before PD-1 in the panel, sufficient tyramide could be deposited to block the PD-1 antigen. If present, this phenomenon might also be diagnosed when the evaluation to singleplex IHC/IF is performed. A useful strategy to determine antibody/fluorophore interference or blocking is the drop controls method to find which one is causing the interference (Surace et al., 2019). To correct this situation, we can increase the primary antibody concentration(s), reduce TSA fluorophore concentration(s), and/or change the order of targets in the panel (Taube et al., 2020).

The second aspect to consider is crosstalk, which is an additional signal from the non-target fluorophore captured by the microscopic system (60, (Arppe et al., 2017). There are commonly recommended practices to cut back this effect; for example, crosstalk is often considerably reduced by choosing fluorophores whose excitation and emission spectra match those of the corresponding channels but minimally overlap those of non-corresponding ones. Alternatively, optimizing the filters of imaging channels, such as the adoption of excitation and emission filters with narrower bandwidths, can very effectively alleviate the crosstalk, although the signal strength might be sacrificed (Tie and Lu, 2020).

## Multispectral Library and Optical Detection

**FIGURE 3:**
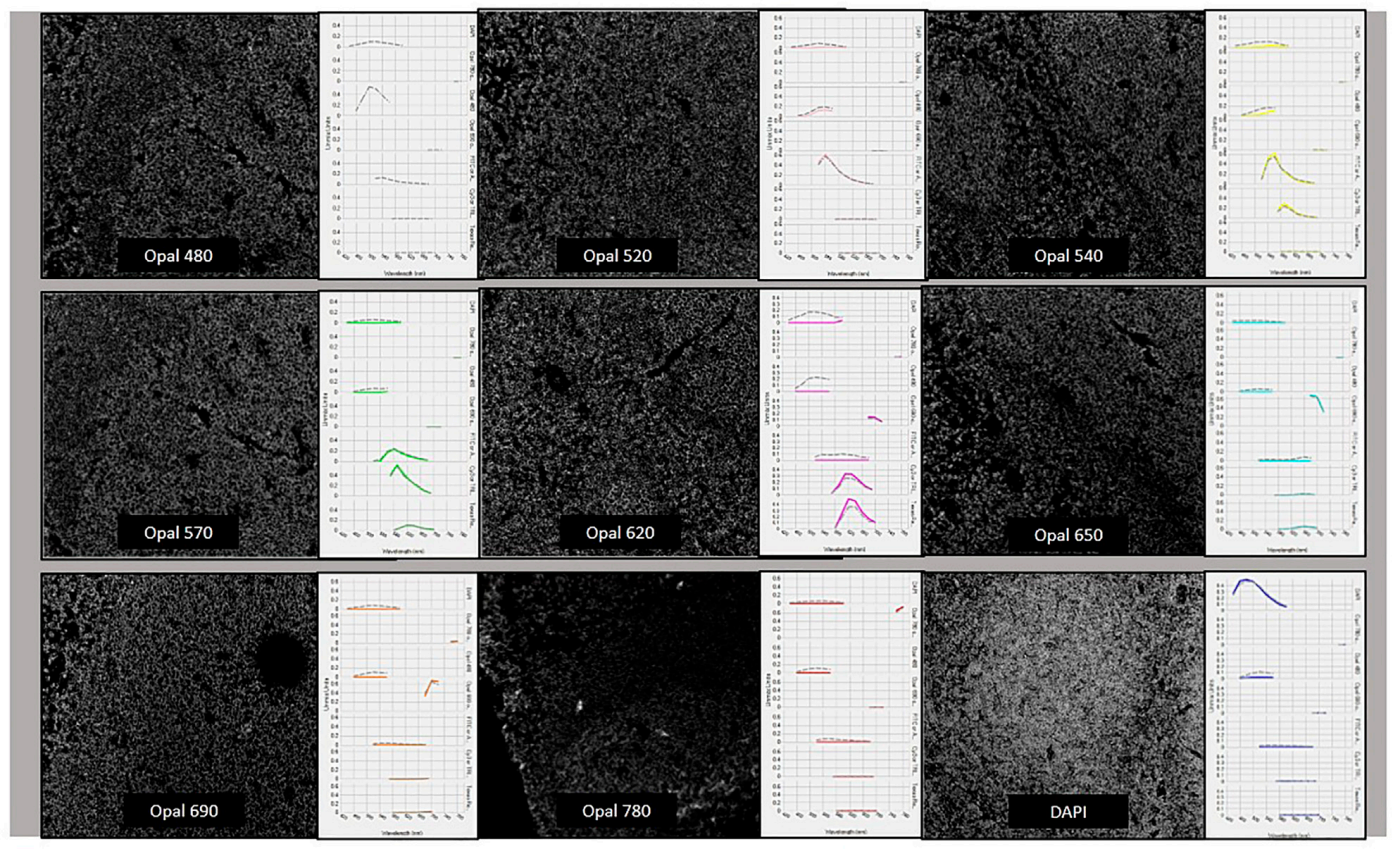
Spectral library creation with the different fluorophores from the Opal-9 kit. Opal 480, Opal 520, Opal 540, Opal 570, Opal 620, Opal 650, and Opal 690, Opal 780, and DAPI were stained, optimized, and validated until we obtained similar dynamic ranges and specific wave peaks as is possible to see in this picture.

Multispectral libraries and their optical detection play an important role in determining the correct extraction of the photophore’s signal according to their fluorescence wavelength. Exposure times need to be set up carefully to maintain a balance of the signal intensity across markers in the panel (Parra et al., 2020). Because we are working with multispectral imaging, additional considerations required for capturing the images include the generation of a spectral library, which will facilitate the discrimination and capture of the individual fluorescence signal using the correct spectra from each fluorophore (Francisco-Cruz et al., 2020b; Parra et al., 2021b; Viratham Pulsawatdi et al., 2020). The creation of the spectral library with a single stained sample for each individual fluorophore corresponding primary antibody will be important for the signal extraction (Figure 3). Also recommended for signal extraction is a marker with a highly prevalent antigen such as CD20, anti-sodium potassium ATPase, or vimentin, as well as rechecking this spectral library regularly depending on whether the scanner system uses a fluorescence bulb or LED light sources for the excitation. Finally, it is important that the signal extraction is from exogenous and endogenous autofluorescence in this methodology (Francisco-Cruz et al., 2020b). Other components in the scanner systems used for acquiring the images that must be considered when choosing the scanner system are multispectral range, fluorescence throughput, automation, and multiplexing capability, among others, to obtain high-quality images (Table 3).

**TABLE 3:** Differences scanners used for multiplex analysis.

Company	Scanner type	Image acquisition and scanning instrument	Corporate location/notes	Resolution	Image extraction	File type	Automatization
Leica Biosystems	BF and FL	Aperio Versa	Illinois, United States	0.468 μm per pixel with ×20 objective. 0.2um at 40x	WS	TIFF, JPEG	Semi-auto and auto
3DHistech	BF and FL	Pannoramic 250 FLASH III	Budapest, Hungary	0.172 and 0.087/0.325 and 0.162 pixels	WS	---	Auto
Ventana/Roche	BF and FL	iScan	United States, International	24-bit true color	WS	TIFF/BIF	Auto and manual
PerkinElmer	MSI (BF and FL)	Vectra/Vectra Polaris	Boston, United States	10× (1.0 μm/pixel), 20× (0.5 μm/pixel) and 40× (0.25 um/pixel)	ROI	QPTIFF, IM3, JPEG, single-layer TIFF, BMP, PNG	Touchless automation with walk-away image acquisition
Olympus America	BF and FL	VS110, Nanozoomer (United States)	Japan, International	0.32 µm/pixel (20×/NA 0.75) - 0.16 µm/pixel (40×/NA 0.95)	WS	Compress images and save images in different file formats	Auto
Zeiss	BF and FL	AxioVision MosaiX	United States, Germany	5× (2.11 µm/pixel), 10× (1.05 µm/pixel), and 20× (0.53 µm/pixel)	WS	AVI, BMP, J2K, JP2, JPG, LSM, MOV. PCT, PCX, PNG, PSD, TGA, TIF, WMF	Auto/manual

BF: bright field; FL: fluorescence: MALDI: matrix-assisted laser desorption/ionization; FOV: field of view; WS: whole section; ROI: region of interest.

## Panel Validation

The final validation of the mIF panel requires the performance of intra-site and inter-site reproducibility studies prior to clinical use (Taube et al., 2020). At this point, the same TMA as used in the antibody validation is an optimal material for validation purposes. The experience with IHC is diverse, according to the marker, without a universal consensus, because each marker is different; in mIF, this knowledge is still being developed. Although automated staining can give us high reproducibility and is recommended for mIF staining, manual staining can be considered to process small quantities of slides at the same time to avoid errors and antibody variability caused by manual manipulation (Parra et al., 2017). Finally, similar strategies as mentioned for antibody validation can be applied for panel validation.

## Analytic Evaluation

Diverse factors could affect the pre-analytical step, as mentioned previously; reagents, autostainer performance, section thickness variation, scanner performance, and change in quality and quantity of the cells between serial sections can influence the mIF analysis (Lee et al., 2020). For an IHC or mIF assay to be considered validated, at a minimum, it must be demonstrated to be accurate and precise, as well as reproducible from an analytic perspective and on pathologist interpretation (Taube et al., 2020).

Marker evaluation is a key aspect of reproducibility. Markers with abundant and specific cell expression, such as CD3, are easy to evaluate and will probably be consistent across serial sections when the expression is evaluated. For markers with variable geographic distribution across tissues and variable tumoral expression, such as PD-L1, reproducibility will be more challenging across serial sections (Parra et al., 2018b). To determine the reproducibility of markers in the mIF panel, we must consider that a group of markers is being evaluated and that those markers have specific cell phenotypes (marker co-expression) across different sections, according to the abundance of specific cell phenotypes (Lee et al., 2020). Marker reproducibility studies are easier in IHC compared with mIF because the evaluation is performed one by one; in mIF, it is harder to evaluate an entire panel using only one method, so the variability is related to the number of markers and their expression is extensive when specific phenotypes are evaluated.

Another drawback for mIF is high inter‐observer variability for the same marker (Gerdes et al., 1984; Vincent-Salomon et al., 2007; Mohammed et al., 2012; Munzone et al., 2012; Cheng et al., 2015; Matsumoto et al., 2015). For instance, Ki‐67 is a widely endorsed marker for a range of cancers Tumeh et al. (2014), but an issue has been raised concerning the reproducibility of IHC for Ki‐67 and the implications of variability in clinical decision-making (Curigliano et al., 2017). Multiple research groups have demonstrated that inter‐observer variability can be negated using digital analysis (Tan et al., 2020). There are different ideas as to the causes of between-pathologist variation; it may be the result of differences in each pathologist’s clinical experience and technological competence (Barnes et al., 2017). In this case, the best approach may be to create a protocol of interpretation, with a consensus across all the groups. It will be useful to perform an objective analysis of each marker, or at least most markers. Having clear examples of false positives or false negatives can also be fundamental.

The selection of representative regions (hot spots) to score, cellular expression or intensity thresholding, binning, overall positive and negative slide rating, and cut-offs are additional challenges to consider in the post-analytic study.

While training and various quality systems have increased pathologists’ scoring repeatability, reproducibility, and accuracy, there is still significant room for improvement (Terrenato et al., 2013; Lin and Chen, 2014; Nielsen, 2015), and the same challenges can arise even in image analysis (Barnes et al., 2017), especially when different laboratories use different image analysis systems. Although computational quantitation using digital image analysis algorithms may improve reader precision performance (Rexhepaj et al., 2008; Ghaznavi et al., 2013; Barnes et al., 2017), it is important to harmonize those systems between laboratories and create protocols to make the data more reproducible.

In digital analyses, the pathologist evaluates a digital image of the glass slide on a computer monitor and uses a computational algorithm to provide a result. The reader selects representative fields of view or regions of interest (ROIs) of the tumor that the algorithm analyzes to yield a score that is intended to represent the whole tumor (Barnes et al., 2017).

As tumors often harbor substantial cellular and spatial heterogeneity, it is essential to perform high-resolution multiplexed analysis across entire tumor sections. Other factors must also be considered when determining whether to select representative ROIs or the entire tissue. Analyzing the entire tumor can be time- and resource-consuming, so it is best is to select areas that are representative of the tumor’s heterogeneity. The analysis of small ROIs or small tissue areas generates important variations and errors in the assessment of tumor and immune markers in cancer (Hofman et al., 2019). Other tumor types may have a higher degree of molecular heterogeneity, which may contribute to outcome (Rizzardi et al., 2016); analyzing a minimum area according to the complexity of each case is the most reasonable solution, but it is also important to have consensus between groups.

Given all of these challenges, laboratories that use mIF should standardize a minimum ROI or tissue area for analysis to generate accurate and reproducible results, considering the bibliographic data already available. The criteria to select areas of analysis should be compared in select representative areas, using the same method for evaluating each marker. In addition, although the algorithm can be locked, it will not always fit all the tumors; thus, it is possible to use an algorithm model as a base and make small changes according to the heterogeneity or type of tumor.

As each sample is complex, it is necessary to determine what factors should be excluded from the analysis (such as necrotic areas, hemorrhagic areas, non-preserved areas, unsatisfactory samples) and to standardize the reasons for exclusion to identify those that are unsatisfactory for mIF; in this way, only the cases without these considerations will be analyzed. The fewer the confusing factors are involved in the results, the easier it will be to standardize the workflow; each analysis could have less interobserver variability.

## Post-Analytic Evaluation

After the pre-analytic and analytic evaluations, it is important to consider inside and outside evaluations. On the basis of our experience with IHC, although internal quality control procedures address daily reproducibility and are fundamental for monitoring performance in individual laboratories, external quality assessment is necessary to compare results from many laboratories by means of an external agency. This step allows the identification of insufficient stains and inappropriate protocols, as well as the identification of possible issues with interpretation (Vyberg et al., 2005; Copete et al., 2011). An external evaluation can provide an objective evaluation of staining results from many laboratories for a given epitope or biomarker, identify the best practice protocols to obtain optimal results, and systematically identify causes of insufficient results (Nielsen, 2015). A similar evaluation is expected to be performed for mIF panels.

Some of the challenges in the pre-analytical and analytical steps have included standardizing the post-analytic component of mIF quantitation, including the interpretation approach, representative region (hot spot) selection, cellular expression, intensity thresholding, and cut-offs. While training and quality systems have increased pathologists’ scoring repeatability, reproducibility, and accuracy, there is still significant room for improvement (Barnes et al., 2017). The experiences of different institutions should be combined in a common effort to standardize tissue scoring.

**FIGURE 4:**
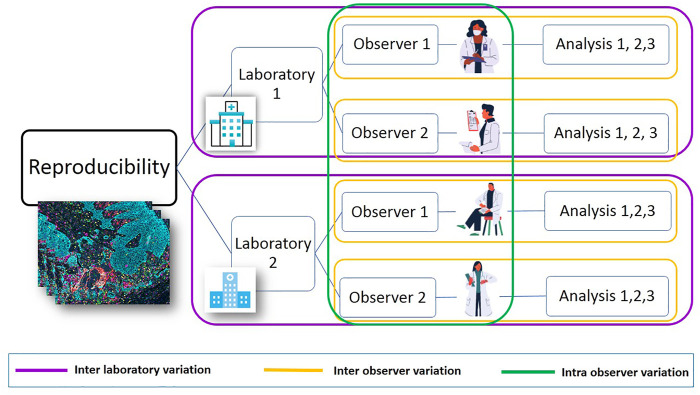
Post-analytic reproducibility study. It is possible to address the flow of post-analytic studies that compare results between two or more sites. Each site can have different observers, and each can analyze slides or projects more than one time using image analysis. This algorithm makes it possible to find variability between laboratories. This workflow is useful for IHC/mIF and other techniques, improving the quality of the final results. It is highly recommended to publish the results and findings.

The final device design and configuration should be verified, including accuracy, technical sensitivity, and specificity and precision (i.e., intra-assay run, inter-assay run, inter-lot variability, inter-reader variability, and inter-instrumentation variability). External analytical validation studies should then be performed to document reproducibility (Figure 4).

Several published reports have described mIF optimization panel methods for solid tumors, but few are fully automated or reproducible for large numbers of samples (Lee et al., 2020) or between multiple institutions. One study described a collaboration between six institutions to develop an automated six-plex assay that is focused on the PD-1/PD-L1 axis and assesses inter- and intra-site reproducibility, on the basis of the percentage of expression by immune cells, in serial sections of tonsils and a lung cancer TMA. This approach improved the reproducibility of PD-L1 and immune cells (Hoyt et al., 2019).

It is necessary to create groups or committees that include experts in mIF from different institutions to generate guidelines and recommendations for staining, optimization, and validation procedures for mIF technology that can help to harmonize this assay across different research laboratories and standardize its clinical application (Table 4). Finally, the goal is to establish only one protocol for all of the institutions that use this technology, making it possible to identify issues even when each lab has its own differences in the items related to pre-analytical and analytical evaluation; however, these differences must not be an excuse to not improve internal protocols or to justify incorrect results.

**TABLE 4:** Stages, challenge, and possible solutions for the best reproducibility of multiplex immunofluorescence panel.

Stage	Problem	Solution	Advantages of solutions	Disadvantages of solutions
Pre-analytical	Antibody specificity and staining	Use of positive and negative controls. Review of publications and experiences related to the Ab	Comparison with standardized process and other experiences	If the Ab does not have a previous protocol, it could result not reproducible
	Type of antibody and preparation	Preference by monoclonal antibody. Use specification of the vendor to prepare it	Better results	Not always is possible to monoclonal antibodies
	Optimization of panels in mIF	Test and work all the markers previously with IHC.	Comparison between IHC and mIF results	Some markers could not stain as the IHC
Analytical	Interpretation of markers	Standardized the interpretation of the most common markers	Interpretation well established	Some markers do not have protocols
	Consideration of areas of analysis and hotspots	Decide the number of representative areas of analysis and avoid select hotspots	Better representativeness	To have the right representative areas not always is possible. Number of ROIs could change depending on the type of tumor
	Type of image analysis	Do not expect to have the same result in all the different types of analysis technique. Consider the differences between software. Each one has its advantage and disadvantages		Experience-dependent
Post-analytical	Variability of intra- and inter-observer	Create protocols. If still persisting some variability, identify the problem	Standardization	Time-consuming and requires additional effort of the collaborators
	External and internal variability	Publish the results of each project. Take the experience of other laboratories to improve	Share knowledge	The new technologies do not have other experiences in other laboratories because they can be expensive

## Conclusions

Reproducibility must be evaluated at each step of the process. Small mistakes could have a large impact on the final results and on reproducibility within and between laboratories. The use of standardized protocols is a good approach to avoid wrong results, poor workflow, or whatever issue could affect the quality and results.
